# Systematically addressing nasal inferior turbinate surgical options

**DOI:** 10.1016/j.bjorl.2021.05.004

**Published:** 2021-05-25

**Authors:** João Carlos Ribeiro, Joana Gonçalves, José Carneiro

**Affiliations:** a Centro Hospitalar UniversitᲩo de Coimbra, Departamento de Otorrinolaringologia, Coimbra, Portugal; b Universidade de Coimbra, Faculdade de Medicina, Coimbra, Portugal

Dear Editor,

The Brazilian Journal of Otorhinolaryngology published a clinically very relevant article from Mehel et al., entitled “Early clinical outcomes of inferior turbinate radiofrequency and lateralization combined with septoplasty”.^1^ We would like to voice a few concerns about the scientific inaccuracies of the article, contributing to the clinical soundness of the investigated topic.

Mehel et al. suggest that both radiofrequency and lateralization of the inferior turbinate present similar outcomes regarding nasal obstruction relief, and that the intervention method should be selected at the discretion of the patient and the surgeon(s).

At first, it should be emphasized that surgical therapy of turbinates’ disorders should always follow an unsatisfactory medical therapy. This has been probably done, but not informed in the article and, less experienced readers may get confused.

After a surgical option has been decided, we must consider nasal obstruction etiology factors. Distinct nasal septum characteristics, columella and nasal valve pathologies will imply different turbinal pathologies along with consequent different outcomes. In addition to these facts, some types of septal deviations will tend to present with a higher rate of sinusitis, implying different clinical outcomes.^2^ We would expect the authors to classify and compare these factors between the radiofrequency and lateralization groups so that such a major bias would have been controlled.

After avoinding these biases, the chosen technique to address inferior turbinate would consider not only the turbinate but also the inferior meatus and medial maxillary sinus wall characteristics. Furthermore, Mehel et al. doesn’t seem to differentiate the bony inferior concha from inferior turbinate mucosa. For example, a patient with a more medially displaced conchal bone without a significant mucosa hypertrophy is more likely to benefit further from a lateralization rather than radiofrequency ablation. Thus, comparing the section of the inferior nasal meatus and the volume of the inferior turbinate should be considered before planning surgery. Conversely, if the medial maxillary sinus wall is more vertical and/or there is a greater mucosal hypertrophy of the inferior turbinate, the radiofrequency would be the method with the best results.

Lastly, and as the authors timely remembered, the lateralization of the inferior turbinate is less costly than the radiofrequency. Why not to perform both techniques? We believe there should have been a third group in the study including patients that undergone both radiofrequency and lateralization of the inferior turbinate.

## Conflicts of interest

The authors declare no conflicts of interest.
